# Tailoring the Degradation Time of Polycationic PEG-Based Hydrogels toward Dynamic Cell Culture Matrices

**DOI:** 10.1021/acsabm.4c00057

**Published:** 2024-03-12

**Authors:** Kathrin Kowalczuk, Valentin D. Wegner, Alexander S. Mosig, Felix H. Schacher

**Affiliations:** † Institute of Organic Chemistry and Macromolecular Chemistry (IOMC), Friedrich-Schiller-University Jena, Lessingstraße 8, D-07743 Jena, Germany; ‡ Jena Center for Soft Matter (JCSM), Friedrich-Schiller-University Jena, Philosophenweg 7, D-07743 Jena, Germany; § Cluster of Excellence Balance of the Microverse, Friedrich Schiller University Jena, Grüne Aue, D-07754 Jena, Germany; ∥ Institute of Biochemistry II, Jena University Hospital, Am Nonnenplan 2-4, 07743 Jena, Germany; ⊥ Center for Sepsis Control and Care, Jena University Hospital, Am Klinikum 1, 07747 Jena, Germany

**Keywords:** polycationic hydrogel, poly(ethylene glycol), 2D cell culture, degradable hydrogels, transforming growth factor β

## Abstract

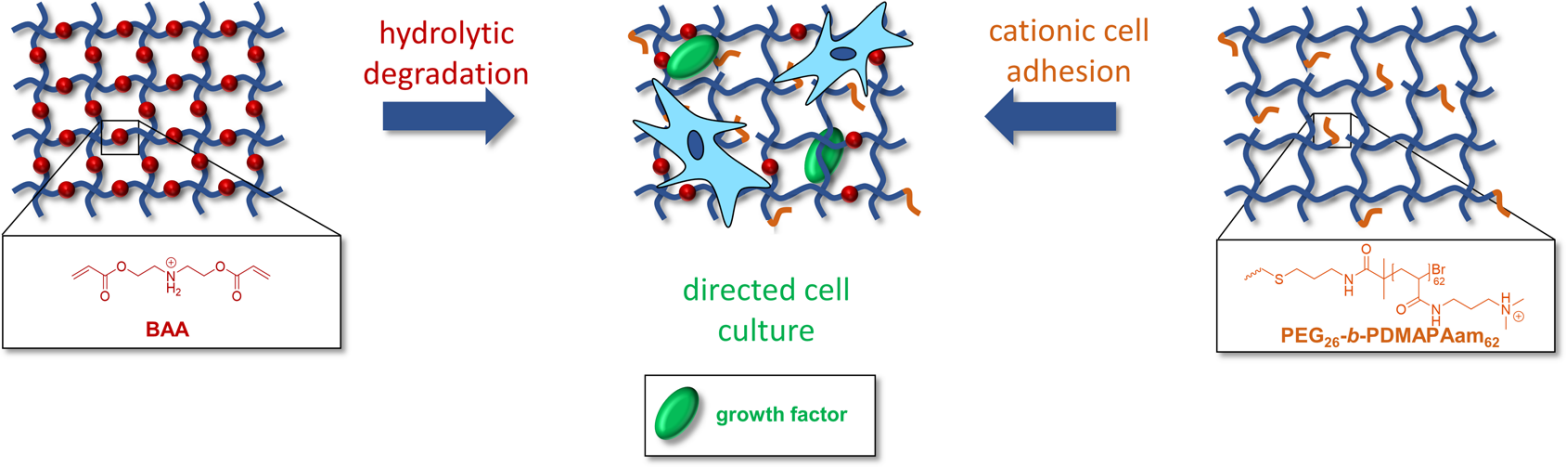

Poly(ethylene glycol)-based (PEG) hydrogels provide an ideal platform to obtain well-defined and tailor-made cell culture matrices to enhance *in vitro* cell culture conditions, although cell adhesion is often challenging when the cells are cultivated on the substrate surface. We herein demonstrate two approaches for the synthesis of polycationic PEG-based hydrogels which were modified to enhance cell-matrix interactions, to improve two-dimensional (2D) cell culture, and catalyze hydrolytic degradation. While the utilization of *N*,*N*-(bisacryloxyethyl) amine (BAA) as cross-linker for *in situ* gelation provides degradable scaffolds for dynamic cell culture, the incorporation of short segments of poly(*N*-(3-(dimethylamino)propyl)acrylamide) (PDMAPAam) provides high local cationic charge density leading to PEG-based hydrogels with high selectivity for fibroblastic cell lines. The adsorption of transforming growth factor (TGF-β) into the hydrogels induced stimulation of fibrosis and thus the formation of collagen as a natural ECM compound. With this, these dynamic hydrogels enhance *in vitro* cell culture by providing a well-defined, artificial, and degradable matrix that stimulates cells to produce their own natural scaffold within a defined time frame.

## Introduction

Poly(ethylene glycol) PEG-based hydrogels as 3D polymeric networks are widely utilized for biomedical applications such as wound dressing,^1,2^ drug delivery,^3,4^ or cell culture.^5,6^ PEG, as a hydrophilic polymer, features good bio- and cytocompatibility, while its highly hydrated networks can emulate the mechanical properties of native tissue.^7^ Despite the lack of cell recognition, the modification with selected biological and chemical additives renders PEG an ideal framework material for cell culture scaffolds which can be modified to target specific cells and mimic the native extracellular matrix (ECM).^8^ Moreover, the commercial availability of PEG building blocks of various molecular weights with different end groups and architectures provides a tunable and low-cost material for tailor-made cell culture matrices.

Cell adhesion to the hydrogel scaffold is generally believed to be the starting point of a successful cell culture. The most common approach is to integrate biological motifs into a synthetic material to mimic the natural environment of the cell.^9,10^ In native tissues, cells adhere to ECM proteins, such as laminin or collagen, by integrin binding. Those surface receptors are connected to the cytoskeleton and show a high affinity to cell adhesion peptide sequences within the proteins.^11^ Among these sequences, l-Arginyl-Glycyl-l-Aspartic acid (RGD) is the most prominent example for the integration into synthetic hydrogels by functionalization with reactive groups such as thiols^12,13^ or acrylates^14,15^ to covalently bind the motif to a given network structure. However, also chemical functionalities can improve the cell adhesion to hydrogels but instead of interacting with the integrin receptors, cationic moieties can favor attachment by attractive electrostatic interactions with the negatively charged cell surface.^16^ Historically this was already exploited since the 1970s when Yavin and Yavin showed that the coating of cell culture dishes with poly(lysine) bearing an amino side group significantly enhances cell adhesion.^17^ Since then, the integration of poly(lysine) into hydrogels has been widely exploited to enhance cell adhesion,^18,19^ but also other polycations are used, such as poly(2-acryloyl trimethylammonium ethyl iodide) (PTMAEA)^20^ or poly(2-(dimethyl amino)ethyl methacrylate) (PDMAEMA).^21,22^

Once cells adhere to the synthetic hydrogel, proliferation, spreading, and migration are highly influenced by the mechanical properties of the scaffold.^23,24^ Since the native ECM is a dynamic environment with continuous remodeling, research gained more and more interest in the imitation of these dynamics by designing degradable hydrogels.^14,25,26^ Similar to addressing cell adhesion, degradable hydrogels often mimic the proteolytic degradation of the native ECM.^27,28^ This can be achieved by the integration of proteinase-sensitive peptide sequences into the network structure. The most prominent proteolytic enzymes, the matrix-metalloproteinases (MMPs), are commonly utilized by functionalization of the respective peptide sequence with reactive end-groups such as thiols on the N- and C-terminus to facilitate the use as cross-linkers.^29^

While this degradation approach depends on the MMP secretion of the cell, inherent and trigger-free degradation can be obtained by the use of hydrolysis-labile cross-linkers.^30,31^ Among them, ester bonds are common, which degrade under physiological conditions by the formation of a hydroxide moiety and a carboxylic acid group. However, degradation of hydrogels by hydrolysis of esters occurs rather slowly at pH 7.4 (>20 days) but can be accelerated to cell culture relevant time scales by introducing cationic functionalities adjacent to the ester bond, which creates a locally controlled and increasingly basic environment.^32−34^ We recently showed that the use of a bisacrylic cross-linker containing a quaternary ammonium group in PEG-based hydrogels accelerates the inherent degradation while providing good cell adhesion.^35^ The hydrogel was formed *in situ* under physiological conditions by thiol–ene click reactions of [PEG-SH]_4_ building blocks, while the degradation time was tailored by different ratios of degradable/nondegradable cross-linking points.

Since studies show that quaternary ammonium groups show the lowest electrostatic cell adhesion among amino functionalities, we herein demonstrate the synthesis of the novel degradable cross-linker *N*,*N*-(bisacryloxyethyl) amine (BAA) bearing a secondary amino functionality for improved cell adhesion. In a second approach, we introduce polycationic stickers based on poly(*N*-(3-(dimethylamino)propyl)acrylamide) (PDMAPAam) by asymmetric block extension of [PEG-SH]_4_, which we recently established for polyampholytic poly(dehydroalanine) (PDha).^36^ The adsorption of the transforming growth factor β (TGF-β) into such polycationic hydrogels stimulated the ECM production of fibroblasts during the degradation of the underlying hydrogel. With this, we designed novel dynamic PEG-based hydrogel scaffolds for improved cell adhesion that provide initial support for the cells while being degraded over time during the formation of a native cell scaffold, thereby providing a material platform for enhanced *in vitro* cell culture.

## Results and Discussion

### Synthesis of [PEG_26_-SH]_3_[PEG_26_-*b*-PDMAPAam_62_] and BAA

For a successful cell culture on PEG-based hydrogels, the cell adhesion is commonly addressed by the incorporation of integrin binding motifs such as RGD.^10,37,38^ The integration of cationic moieties provides an alternative binding possibility through electrostatic interactions and simultaneously provides functionalities to (reversibly) bind charged molecules such as proteins or growth factors to design a versatile cell culture scaffold. Hence, we synthesized the bisacrylic cross-linker BAA according to Xun et al.^39,40^ In addition to the enhanced cell adhesion due to the secondary amino group, the positioning of ester bonds in close proximity creates an inherent, self-degrading cross-linker which provides both cell adhesion and dynamic mechanical environment.

**Figure 1:**
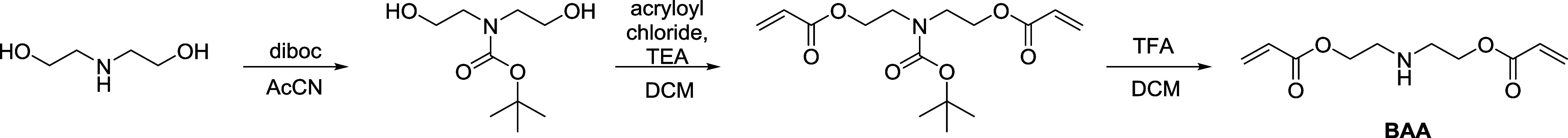
Synthetic route toward the degradable cross-linker BAA.

Therefore, the amino group of diethanolamine was first protected by boc for the subsequent esterification of the alcohol groups (Figure 1). After the protection reaction, the ^1^H NMR spectrum showed the additional signal of the boc group at 1.48 ppm (Figure S1A). Subsequently, the diacrylate was formed by esterification with acryloyl chloride and triethylamine (TEA) as a base, which resulted in the appearance of three signals in the ^1^H NMR spectrum in the double bond region at 6.43, 6.13, and 5.86 ppm, respectively (Figure S1B). In the last step, the boc group was removed with TFA to obtain the free amino functionality. Here, the removal of excess TFA by basic extraction was carried out carefully to prevent hydrolysis of the ester bonds. The successful deprotection was proven by the complete disappearance of the signal at 1.48 ppm in the ^1^H NMR spectrum (Figure S1C) and BAA was obtained with a yield of 78% as a colorless oil.

**Figure 2:**
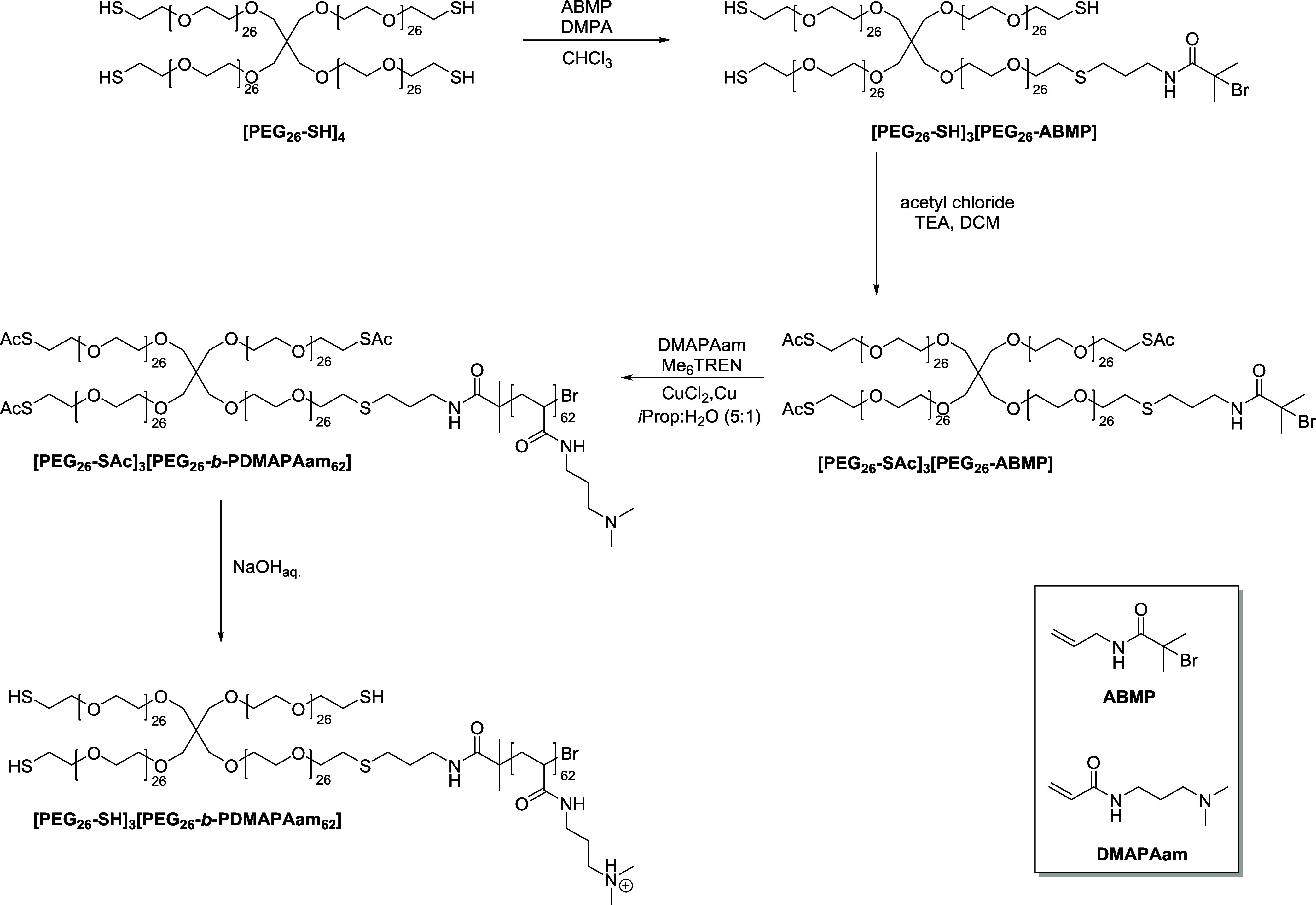
Synthetic route toward the asymmetric star-shaped block copolymer [PEG_26_-SH]_3_[PEG_26_-*b*-PDMAPAam_62_].

In addition to the amino-containing cross-linker BAA, we synthesized an asymmetric star-shaped block copolymer containing short segments of PDMAPAam to increase the charge density in the hydrogel network and its adsorption capacity for negatively charged molecules. Recently, we established a synthetic pathway for asymmetrical block extension of [PEG_26_-SH]_4_,^36^ which was herein used to integrate PDMAPAam (Figure 2).

**Figure 3:**
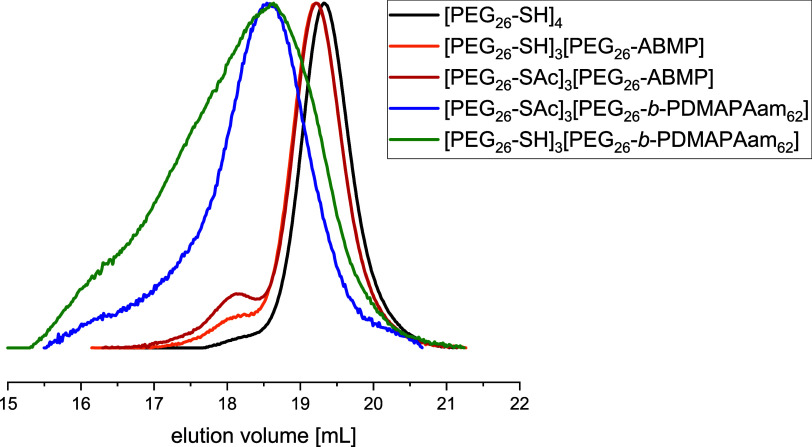
Normalized SEC traces (in DMAc + 0.21 wt % LiCl) of the starting material [PEG_26_-SH]_4_, the asymmetric block copolymer [PEG_26_-SH]_3_[PEG-*b*-PDMAPAam_62_], and the respective intermediates.

First, *N*-allyl-2-bromo-2-methylpropanamide (ABMP) was conjugated to [PEG_26_-SH]_4_ via photoinitiated thiol–ene reaction in a thiol:ene ratio of 4:1 to provide an initiation site for the block extension while maintaining free thiol groups for subsequent network formation. The successful functionalization was determined by ^1^H NMR spectroscopy (Figure S2A) by comparing the integrals of the PEG backbone signal at 3.72 ppm to the signal of the methyl groups of ABMP at 1.95 ppm. One has to note that the distribution of the initiation site is statistical, and hence also 2-fold, 3-fold, and 4-fold functionalized PEG building blocks are possible.^36^ However, the narrow and monomodal distribution of the SEC trace after block extension indicates that the resulting inhomogeneities can be neglected. After the initiator site was successfully conjugated, the remaining thiol groups were protected with acetyl chloride under basic conditions to avoid side reactions during the polymerization. Successful protection was determined by comparing the integrals of the ^1^H NMR signal of the polymer backbone and the signal of the protecting group at 2.38 ppm (Figure S2B). During the synthesis of the macroinitiator [PEG_26_-SAc]_3_[PEG_26_-ABMP], the second distribution in the SEC traces (Figure 3) slightly increased, which we assign to the formation of dimers by oxidative disulfide formation. However, this side reaction can be neglected since it also deactivates the thiol groups similarly to the protective group, and potential disulfide bonds were reduced prior to the hydrogel synthesis with TCEP.

The obtained macroinitiator was used for the polymerization of DMAPAam via single electron transfer living radical polymerization (SET-LRP) to obtain a relatively short segment while maintaining control over the dispersity (*Đ*) and molecular weight (*M*_n_). Due to the low reactivity of acrylamides, we used tris[2-(dimethylamino)ethyl]amine (Me_6_TREN) as a highly reactive ligand and a solvent mixture of water/*iso*-propanol (1:5 v/v) to increase the polarity. Under these conditions, the ^1^H NMR spectrum showed a degree of polymerization of 62 through the comparison of the integrals of the signal of the PEG backbone and the methyl groups of PDMAPAam at 2.13 ppm (Figure S3A). The SEC trace shows a clear shift toward lower elution volumes compared to the macroinitiator and a moderately low dispersity of 1.2 (Figure 3).

The obtained asymmetric block copolymer [PEG_26_-SAc]_3_[PEG_26_-*b*-PDMAPAam_62_] was subsequently deprotected with NaOH_aq._ to reactivate the thiol groups for network formation. The appearance of monomodal SEC traces supports our assumption that the amide bond of ABMP remained stable during the deprotection (Figure 3), so no significant cleavage of the second block occurred. Moreover, the ^1^H NMR spectrum proved the SEC data by retaining the integrals of the PDMAPAam signals subsequent to the deprotection. Additionally, the ^1^H NMR spectrum showed a significant shift toward a lower field for the signals of PDMAPAam, which is due to the protonation of the amino group (Figure S3B), leading to the broadening of the SEC trace due to increased interactions with the column material. Thus, we were able to synthesize the asymmetric block copolymer [PEG_26_-SAc]_3_[PEG_26_-*b*-PDMAPAam_62_] that can be integrated into PEG-based hydrogels to promote cell adhesion.

### Hydrogel Formation

**Figure 4:**
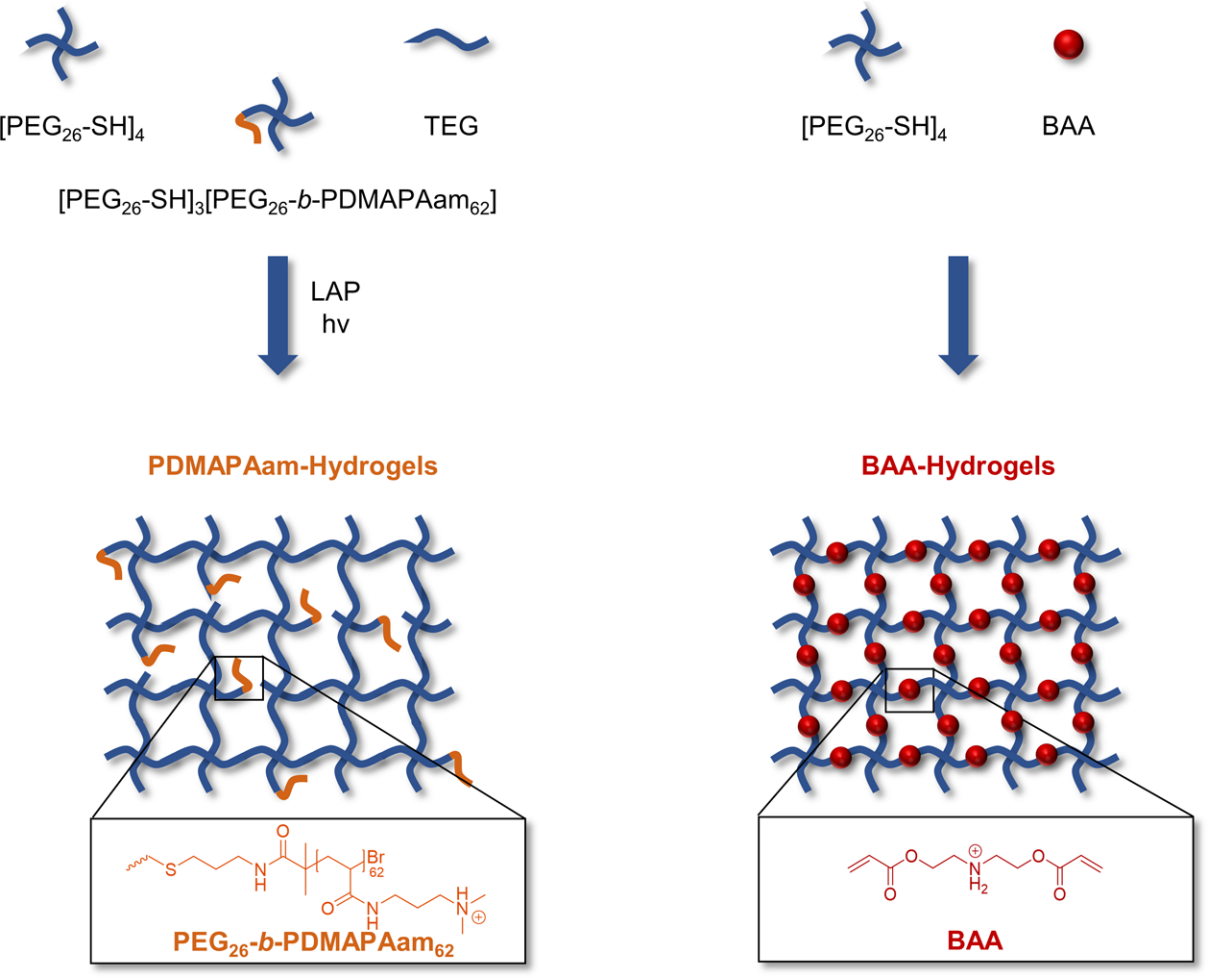
Synthetic route toward polycationic PDMAPAam-hydrogels (left) and degradable and cationic BAA-hydrogels (right).

For the formation of polycation, PEG-based hydrogels, either BAA or PDMAPAam, were utilized for hydrogel synthesis (Figure 4). To synthesize PEG-based hydrogels promoting cell adhesion and with tunable degradation, we used BAA as a cross-linker for [PEG_26_-SH]_4_ in a step growth thiol–ene reaction. Due to the high reactivity of the bisacrylic cross-linker, the network formed *in situ* within 1 min at room temperature without the demand of an additional initiator via a Michael addition when solutions of both components in PBS were mixed. This provides a straightforward and biocompatible hydrogel synthesis for use in cell culture applications.

**Figure 5:**
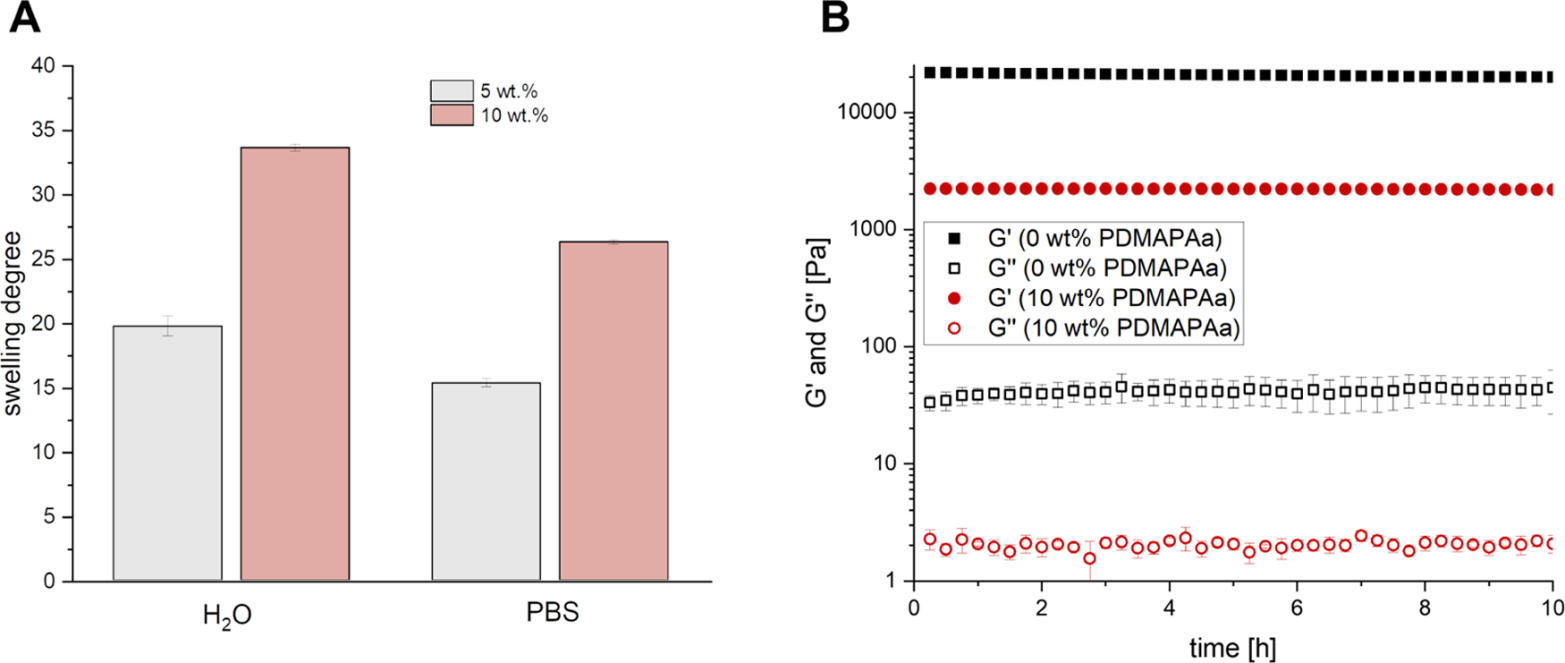
(A) Swelling degree of PDMAPAam-containing hydrogels in DI-water and PBS. (B) Time sweep showing storage modulus (*G*′) and loss modulus (*G*″) of pristine PEG hydrogels and hydrogels with a PDMAPAam content of 10 wt %.

On the other hand, we integrated the asymmetric star-shaped block copolymer [PEG_26_-SAc]_3_[PEG_26_-*b*-PDMAPAam_62_] into a PEG-based hydrogel (Figure 4). To form a nondegradable but cationic hydrogel tri(ethylene glycol) divinyl ether (TEG) was used as a cross-linker in combination with the water-soluble and biocompatible photoinitiator lithium phenyl-2,4,6-trimethylbenzoylphosphinate (LAP). Since the introduction of PDMAPAam causes defects in the hydrogel structure which decreases the mechanical stability of the hydrogel, a combination of [PEG_26_-SH]_4_ and [PEG_26_-SAc]_3_[PEG_26_-*b*-PDMAPAam_62_] in different ratios was tested. The hydrogels lost their form stability when more than 10 wt % of PDMAPAam were introduced to the network, hence the swelling behavior of PDMAPAam-hydrogels with 5 and 10 wt % was investigated (Figure 5A). The increased swelling with increasing concentration of the copolymer is assigned to the above-mentioned raised number of defects which results in an overall larger average mesh size. Moreover, the hydrogels show increased swelling when DI water is used instead of PBS. This is related to the polyelectrolyte effect that causes electrostatic repulsion in between protonated amino groups, leading to stretching of the entangled PDMAPAam segments within the meshes, while the addition of salts with PBS provides counterions for electrostatic shielding.^41^ Since we aimed for stable hydrogels while providing a high content of PDMAPAam we used for further experiments a final PDMAPAam concentration of 10 wt %. For this hydrogel, the mechanical properties were investigated via rheology. The PDMAPAam-containing hydrogel shows a decreased storage modulus (*G*′) of 2.2 ± 0.1 kPa compared to a pristine PEG hydrogel formed under the same conditions, which exhibits a storage modulus of 20.8 ± 0.5 kPa (Figure 5B) which can also be assigned to the formation of a nonideal network structure. However, we could show that the introduction of the asymmetric copolymer [PEG_26_-SAc]_3_[PEG_26_-*b*-PDMAPAam_62_] affects the mesh size and cross-linking density resulting in different mechanical properties.

The strategies shown herein to introduce cationic moieties into the PEG-based hydrogels to improve cell adhesion and adsorption capability resulted in a change in the hydrogel properties toward fully degradable hydrogels in the case of hydrogels cross-linked with BAA. To further increase the charge density while maintaining the degradability, both strategies were combined in PDMAPAam/BAA hydrogels. However, the high selectivity between cells and substrate did not lead to successful cell cultivation of the herein used cell lines on the combined hydrogels so far. However, we are confident that the combination of both strategies can provide very attractive 2D cell culture matrices for future studies.

### Degradation Kinetics

**Figure 6:**
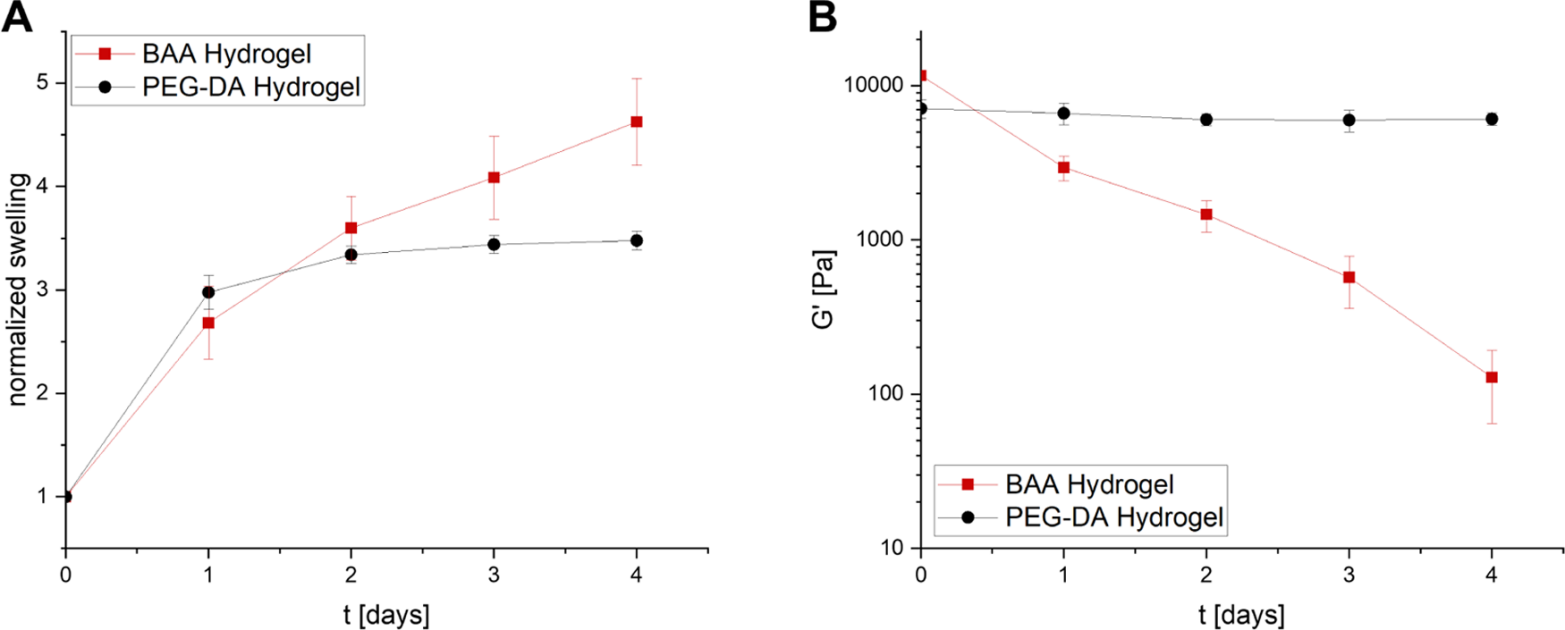
(A) Normalized swelling of degradable BAA-hydrogels and nondegradable hydrogels cross-linked with PEG-DA in PBS. (B) Storage modulus (*G*′) of degradable BAA-hydrogels and stable hydrogels cross-linked with PEG-DA in PBS.

To mimic the dynamics of the native ECM, BAA was used as an inherently degradable cross-linker for PEG-based hydrogels as a cell culture matrix. We recently showed that amino-containing bisacrylic cross-linkers show accelerated degradation due to a local basic environment.^35^ Hence, we investigated the dynamics of BAA hydrogels by monitoring the swelling and storage modulus upon degradation in PBS. For both methods, the BAA-hydrogels showed form stability for up to 4 days. During degradation, the mesh size increased, which resulted in an increased water uptake; hence, the normalized swelling increased (Figure 6A). Following this, both the storage modulus and the stiffness decreased due to the increased water uptake, leading to a softening of the hydrogel (Figure 6B). As a comparison, both measurements were carried out with a PEG-diacrylate cross-linker which does not show hydrolysis on this time scale.

**Figure 7:**
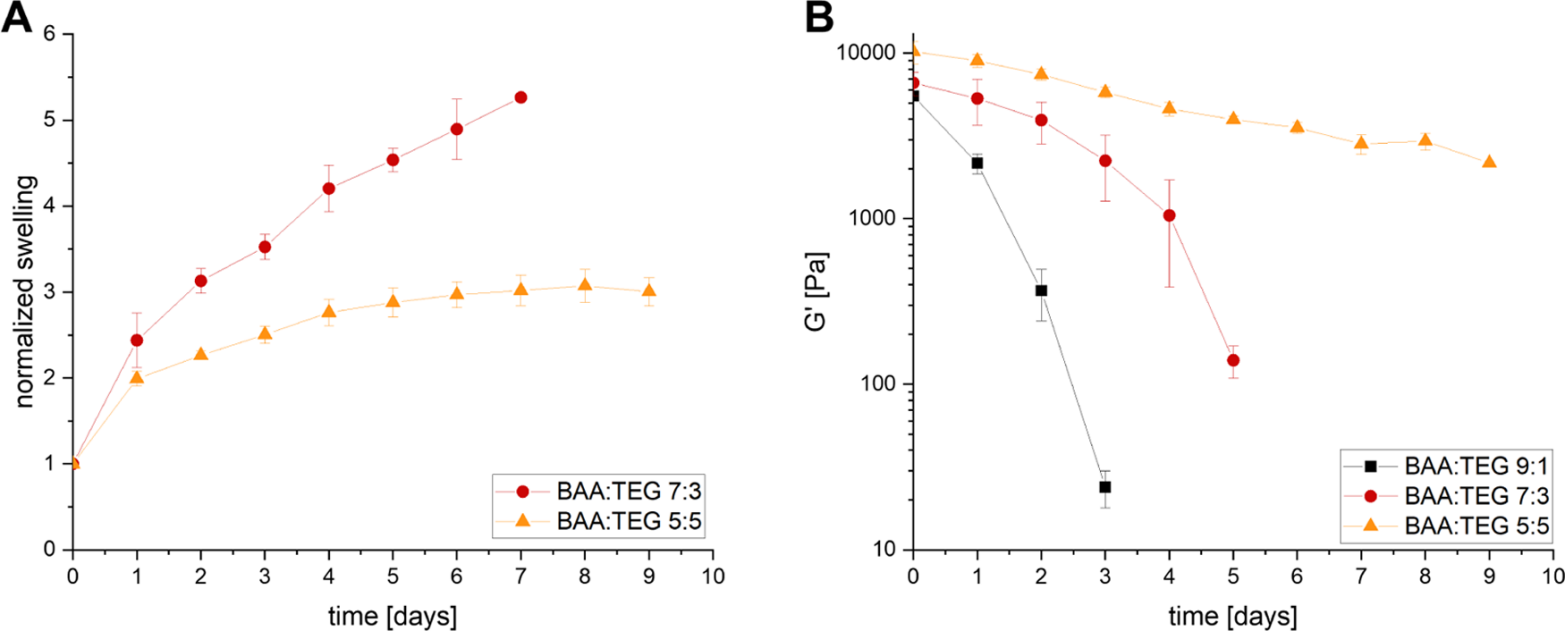
(A) Normalized swelling of degradable hydrogels with different BAA/TEG ratios in DMEM. (B) Storage modulus (*G*′) of degradable hydrogels with different BAA/TEG ratios in DMEM.

Since earlier work showed that the degradation kinetics of bisacrylic cross-linkers are accelerated under cell culture conditions with Dulbecco’s modified Eagle medium (DMEM),^35^ we combined the degradable cross-linker BAA with nondegradable TEG to manipulate the degradation dynamics. The rheological monitoring of hydrogels with BAA/TEG ratios of 9:1, 7:3, and 5:5 illustrates slower degradation kinetics with an increasing TEG content (Figure 7B). While a ratio of 9:1 resulted in a loss of form-stability within 3 days, the stability could be extended to 6 days with a ratio of 7:3. If a 5:5 ratio was used, the hydrogels showed partial degradation in DMEM within the first 6 days, followed by a stable plateau. Moreover, the increasing TEG ratio resulted in an increase of the initial storage modulus from 5.5 ± 0.3 kPa in the case of a 9:1 ratio to a storage modulus of 10.2 ± 1.6 kPa for a 5:5 ratio. This is caused by the reduced hydrophilicity of uncharged TEG in comparison to cationic BAA. The trend of the rheological degradation kinetics was confirmed by measurements of the normalized swelling. Since the hydrogels with a 9:1 ratio do not show degradation on a cell culture-relevant time scale, we measured the normalized swelling of hydrogels with a BAA/TEG ratio of 7:3 and 5:5. We could confirm the results from rheology resulting in a final normalized swelling of 5.3 ± 0.0 for a ratio 7:3 at day 7 and 3.00 ± 0.16 for a ratio of 5:5 at day 9 before the loss of form stability (Figure 7A).

By variation of the ratio between degradable and nondegradable cross-linkers, we were thus able to tune the initial mechanical properties and the degradation kinetics to design tailor-made, dynamic cell culture scaffolds. Thus, a BAA/TEG ratio of 7:3 was used for further cell culture applications to provide a gradual degradation over a cell culture-relevant time scale while maintaining full degradation of the scaffold.

### Cell Culture

In previous work, we showed that PEG hydrogels only cross-linked with TEG lack cell adhesion, leading to apoptosis, which was successfully avoided by the introduction of charged moieties such as amino groups.^35^ To test the suitability of the novel polycationic PEG-based hydrogels as cell culture matrices and evaluate their suitability as directional scaffolds, fibroblasts of different cell lines (3T3-J2 and LX-2) were cultivated on top of the hydrogels. While 3T3-J2 cells showed confluent cell growth on hydrogels cross-linked with BMSAB and BDMAI, which share structural similarities with BAA,^35^ LX-2 cells show an increased release of pro-collagen I when triggered with TGF-β.^42^

**Figure 8:**
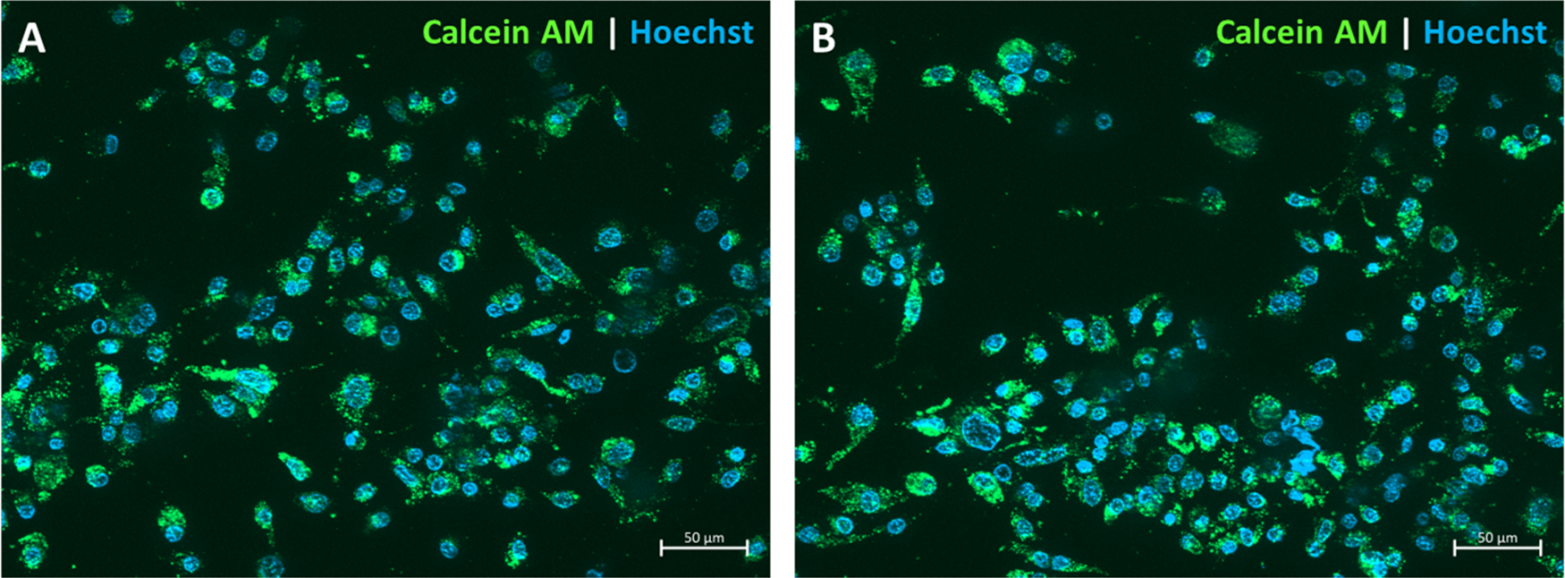
Fluorescence images of hydrogel-specific cell growth: (A) 3T3-J2 cell growth on PDMAPAam hydrogels and (B) LX-2 cell growth on BAA hydrogels; after 4 days of cultivation, the cells were stained with the viability dye Calcein AM and Hoechst to stain the nuclei.

Cultivation of 3T3-J2 and LX-2 cells on the above-described BAA, PDMAPAam hydrogels showed hydrogel-specific cell growth. Whereas 3T3-J2 cells were able to grow on vitronectin-coated PDMAPAam hydrogels, they were not able to grow on the BAA hydrogels. On the other hand, LX-2 cells were only able to grow on BAA-containing hydrogels. We assign this specificity to a lack of cell adhesion since the requirements of adhesion differ widely between cell types and cell lines. Cell attachment on surfaces depends on a wide range of factors, such as rigidity, surface charge, and charge accessibility.^43^ Once cells are detaching from ECM, they enter the state of anoikis, followed by apoptosis.^44^ This is the reason that distinct cell growth is observable. For better comparison of the cell lines and investigation of the influence of the cell-matrix interactions on the stimulation, we additionally synthesized a mixed PDMAPAam/BAA hydrogel in order to enable the growth of both cell lines. Interestingly, the combination of both types of hydrogels in the mixed PDMAPAam/BAA prevented cell growth within the scope of this study. Figure 8 shows exemplary images of the specific cell types growing on the respective hydrogels and stained for their nuclei with Hoechst in blue and Calcein AM as a viability dye, showing cell-specific confluent growth.

### Stimulation of Extracellular Matrix Protein Deposition by TGF-β

While the polycationic PEG-based hydrogels show highly cell-specific suitability as cell culture matrices, both BAA and PDMAPAam hydrogels were tested on their ability to direct specific cellular behavior by the adsorption of a growth factor into the scaffold. TGF-β is a suitable growth factor for both 3T3-J2 and LX-2 cells, and such fibroblasts are relevant cells to study the effects of increased extracellular matrix protein deposition, a hallmark of fibrosis.^42^ Hereby, the reversible adsorption of TGF-β inside the mesh of the hydrogels enables a gradual release of the growth factor over time. Due to the complexity and size of the growth factor, various binding mechanisms, such as hydrogen bonding and electrostatic attraction with negatively charged domains, can influence the adsorption.

**Figure 9:**
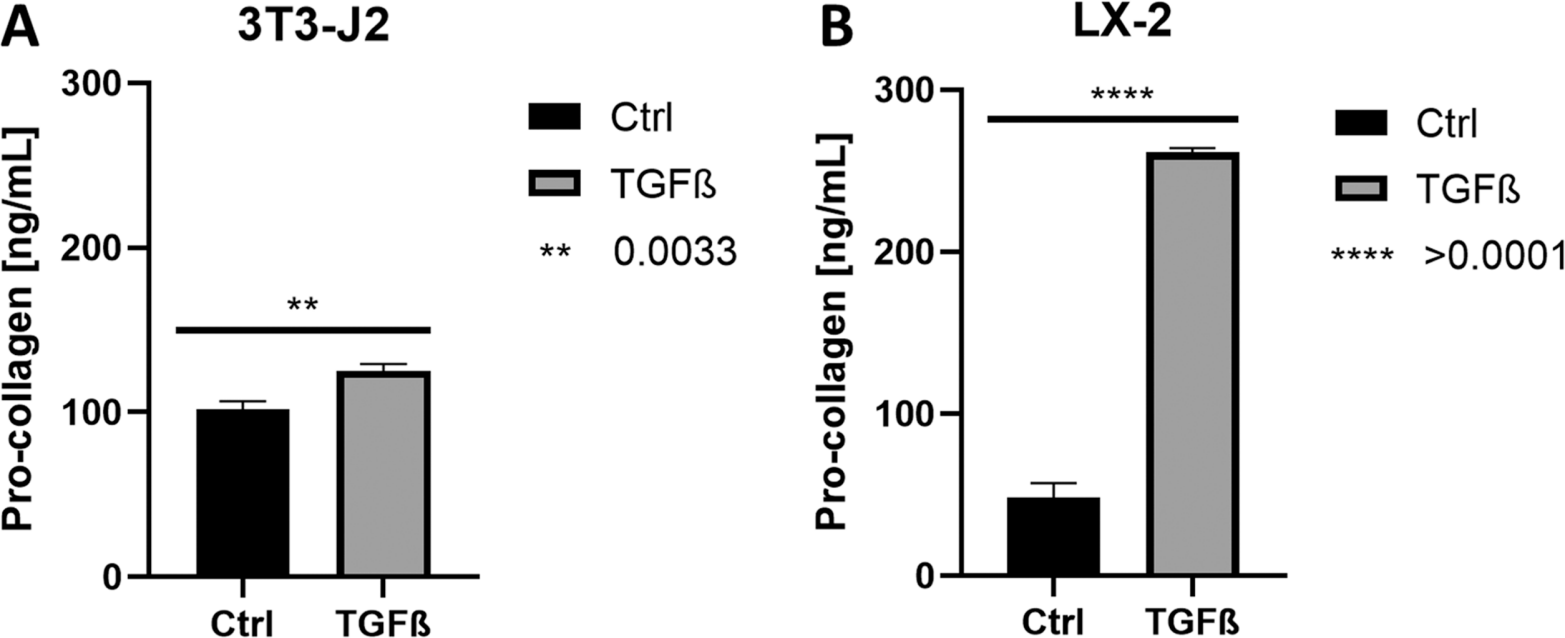
Results of EIA of pro-collagen measured in the cell culture supernatant of 3T3-J2 cell cultivated on PDMAPAam hydrogels (A) and LX-2 cells cultivated on BAA hydrogels (B) after 4 days with and without TGF-β stimulation (each measured value represents cell culture supernatant from a separate hydrogel; significance was determined via *t*-test).

To incorporate TGF-β, the hydrogels were soaked with a TGF-β solution for 3 days, followed by cell seeding and cultivation for 4 days with a daily medium exchange. The successful adsorption of the growth factor was determined indirectly by the quantification of pro-collagen I and collagen I as exemplary ECM components released during fibrosis. Since collagen I production can be detected as early as 2 days after initial stimulation and is highly dependent on the cell type,^45^ quantification was carried out after 4 days to yield reliable results. The successful stimulation was assessed in two stages: First, through measurement of pro-collagen I secreted to the cell culture supernatant, and second, through immunofluorescence staining of collagen I released by TGF-β -stimulated cells at the top of the hydrogels. The pro-collagen I enzyme immunoassay (EIA) (Figure 9) showed a significant increase in the production of pro-collagen I for both cell types, 3T3-J2 and LX-2, when stimulated with TGF-β compared to cells grown on hydrogels without TGF-β functionalization. The increase and total amount of pro-collagen I released by LX-2 cells was 5-fold higher compared to nonstimulated cells.

**Figure 10:**
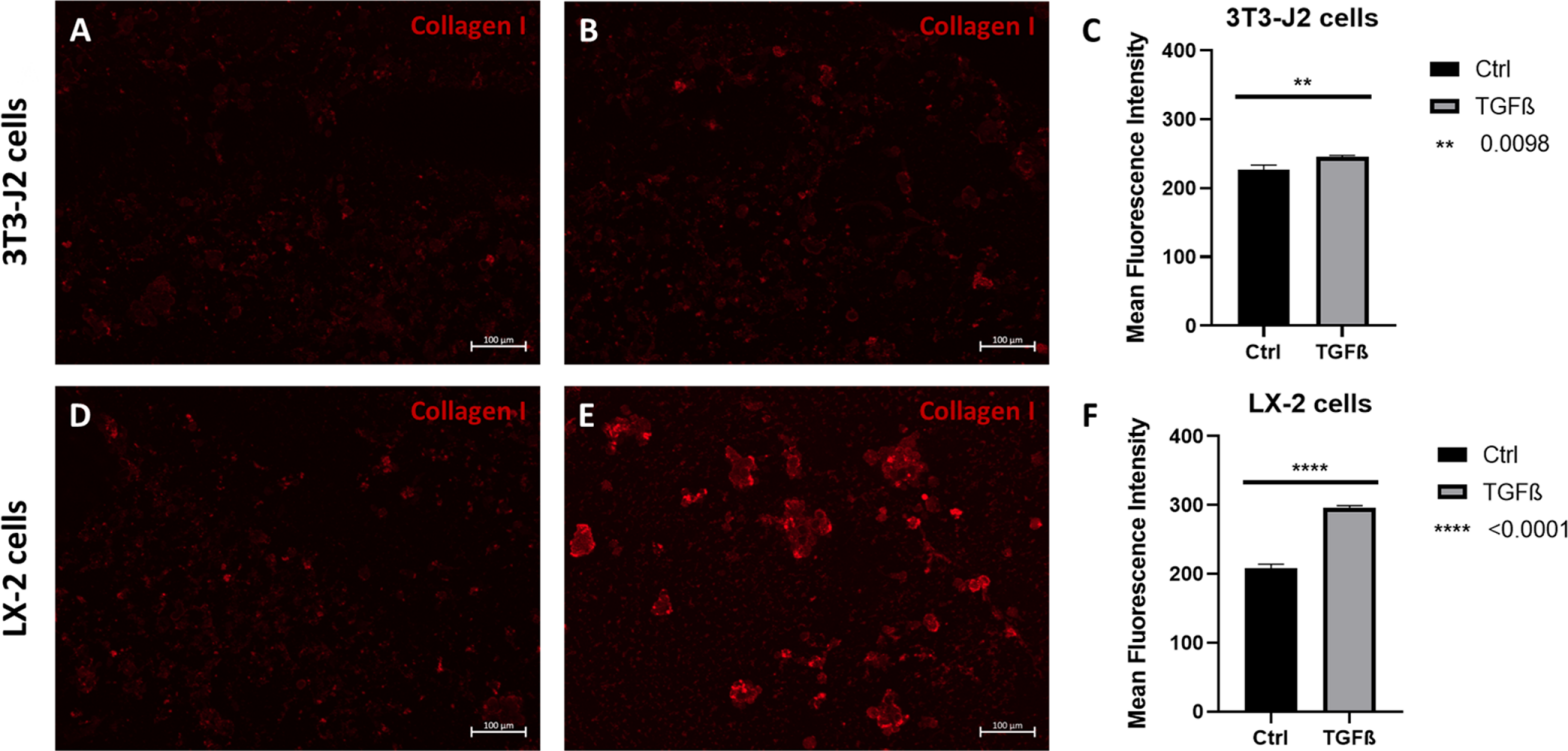
Fluorescence images of produced collagen I (red) of 3T3-J2 cell cultivated on PDMAPAam hydrogels with (A) and without (B) TGF-β and of LX-2 cells cultivated on BAA hydrogels with (D) and without (E) TGF-β after 4 days; (C) and (F) mean fluorescence intensity of the fluorescence images (for each condition, *n* = 3 images were analyzed; significance was determined via *t*-test).

Similar to the pro-collagen I production, the cells showed a significant increase of collagen I deposited at the top of the hydrogel, verifying the successful stimulation of both cell types through TGF-β bound to hydrogels that act as a growth factor reservoir. The deposited amount of collagen I was determined through immunofluorescence staining, fluorescence microscopy, and quantification of the mean fluorescence intensity (Figure 10). Again, stimulated LX-2 cells showed a higher release of collagen-I compared to 3T3-J2 fibroblasts confirming the cell type-specific stimulation.^42^

In summary, both cell types showed a significant increase in collagen I release, whereas collagen I production by LX-2 cells was the highest. Besides the higher susceptibility of LX-2 to TGF-β stimulation, the degradability of BAA hydrogels potentially results in an increased accessibility of the growth factor. However, a direct comparison was not possible due to cell-type-specific growth of both cell lines on the different hydrogels.

The successful cultivation and subsequent stimulation of fibroblasts on the polycationic PEG-based hydrogels opens up the possibility of using hydrogel scaffolds in an improved fibrosis model, in which the hydrogels gradually release TGF-β as a stimulus without continuously adding the factor to the cell culture medium.

## Conclusions

We herein described the successful cultivation of fibroblasts and their stimulation with TGF-β through polycationic PEG-based hydrogels. The synthesis of the degradable, bis-acrylic cross-linker BAA and the asymmetric block copolymer [PEG_26_-SAc]_3_[PEG_26_-*b*-PDMAPAam_62_] provided two different approaches toward the integration of positively charged moieties into PEG-based hydrogels. With that, we were able to address the lack of cell adhesion known for PEG hydrogels and create a dynamic cell culture scaffold. The inherent degradation of hydrogels cross-linked with BAA was investigated by swelling kinetics and the monitoring of the storage modulus and showed full degradation within 4 days in PBS. Due to the accelerated degradation in DMEM, BAA was mixed with the non-degradable cross-linker TEG which resulted in a tunable degradation time depending on the BAA/TEG ratio. In the case reported here, we used a BAA/TEG ratio of 7:3 with a degradation time of 6 days.

The cultivation of 3T3-J2 cells and LX-2 cells on PDMAPAam and BAA hydrogels resulted in highly hydrogel-specific cell growth unless both hydrogels bear cationic amino moieties, showing the complexity of cell-matrix interactions. This resulted in the successful cell cultivation of 3T3-J2 cells on PDMAPAam hydrogels, while LX-2 showed cell growth on BAA hydrogels. However, combining both approaches in mixed PDMAPAam/BAA hydrogels did not result in cell growth of any of both cell types. Besides successful cultivation of the cell lines, we could demonstrate the direction of cellular behavior through the adsorption of TGF-β into the hydrogels. By the gradual release of the growth factor, the stimulation of collagen I synthesis and extracellular deposition was achieved, whereby LX-2 cells showed a 5-fold increase in collagen production compared to hydrogels without TGF-β functionalization.

With that, the herein-presented hydrogels not only provide well-defined and artificial structural support but also act as a reservoir for signaling molecules. This will potentially allow the use of an improved fibrosis model in the future, as well as investigations of diverse cellular behavior by integration of other signaling molecules.

## Materials and Methods

### Materials

Allylamine (98%), α-bromoisobutyryl bromide (98%), 2,2-dimethoxy-2-phenylacetophenone (99%), CuCl_2_ (99%), Cu powder (99.999%), tri(ethylene glycol) divinyl ether (98%), acryloyl chloride (97%), tris[2-(dimethylamino)ethyl]amine (97%), and lithium phenyl-2,4,6-trimethylbenzoylphosphinate (≥95%) were purchased from Sigma-Aldrich. Trifluoroacetic acid (≥99.9%) and tris(2-carboxyethyl)phosphine hydrochloride (≥98%) were purchased from Roth, and acetyl chloride (>98%) was purchased from Alfa Aesar. *N*-[3-(Dimethylamino)propyl]acrylamide (>98.0%) was purchased from TCI, and triethylamine (>99%) was purchased from CHEMSOLUTE. Di-*tert*-butyldicarbonate was purchased from Carbolution Chemicals, and [PEG-SH]_4_ was purchased from JenKem Technology. All chemicals were used as received, and the stabilizer of acryloyl chloride and *N*-[3-(dimethylamino)propyl]acrylamide were removed by filtration over aluminum oxide.

Mouse fibroblasts 3T3-JS were purchased from Kerafast. The LX-2 cells, as well as all cell culture components, DMEM containing 1 g/L glucose, FBS, glutamax, pyruvate, and penicillin/streptomycin solution, were all purchased from Sigma-Aldrich. Vitronectin was purchased from Stemcell. For the fluorescence microscopy, the Hoechst staining and Calcein AM dye were purchased from Thermo Fisher Scientific. The rabbit collagen I antibody was purchased by Abcam, and the secondary donkey antirabbit AF647 antibody was purchased by Jackson Immuno Research.

The procollagen type I C-peptide was measured from the cell culture supernatant with an enzyme immunoassay (EIA) from Takara.

### Instruments

^1^H NMR spectra were performed on a Bruker AC 300 MHz using D_2_O as the solvent at a temperature of 298 K. The spectra were referenced by using the residual signal of the deuterated solvent.

An Agilent 1200 system equipped with an LC-20AD pump, a SIL-20AHT autosampler, and a PSS GRAM analytical 10 μm column (guard/30/1.000 Å) was used for SEC measurements. DMAc + 0.21 wt % LiCl was used as an eluent at a flow rate of 1 mL min^–1^. The column oven (Techlab) was set to 40 °C and signals were detected by using a RID (G1362A) detector. The system was calibrated using PSS PEG (400 to 1,000,000 g·mol^–1^) standards.

Ultrasonication was performed by using an ElmaSonic S30H ultrasonic unit.

UV irradiation was carried out in a UVACUBE 100 (Hoenle UV Technologies) equipped with a 100 W mercury lamp and placed on a stirring plate.

Potentiometric pH titrations were carried out using an OMNIS Advanced Titrator (Deutsche METROHM Prozessanalytik GmbH & Co. KG, Filderstadt, Germany) equipped with a magnetic stirrer, a Pt1000 temperature sensor, and a dosing module. For pH detection, an ECOTRODE plus pH-glass electrode. The compounds were dissolved in 0.1 M NaOH to obtain a starting concentration of 20 mg mL^–1^ and subsequently titrated with 0.1 M HCl.

Rheology measurements were carried out on an MCR 302e modular compact rheometer (Anton Paar, Austria) equipped with a Peltier temperature device P-PTD220/AIR and with a plate–plate measuring system PP20. To avoid solvent evaporation, a solvent trap was used.

Microscopic images were taken on an Axio Observer 5 in combination with an ApoTome 2 of Zeiss AG and quantified using CellProfiler software.

The absorbance for the procollagen EIA was performed at 450 nm on a spectrophotometer 1510 of ThermoFisher Scientific.

### Synthesis of *N*-(*tert*-Butoxycarbonyl)diethanolamine

Diethanolamine (5 g, 1 equiv) was dissolved in AcCN (100 mL). Di-*tert*-butyl dicarbonate (10.3 g, 1 equiv) was added dropwise, and the solution was stirred for 3 h at room temperature. Afterward, the solvent was removed under reduced pressure to obtain the product as a colorless oil (9.8 g, 100%).

^1^H NMR (300 MHz, CDCl_3_, δ): 3.81 (t, *J* = 5.0 Hz, 4H), 3.45 (dd, *J* = 9.6, 4.4 Hz, 4H), 1.48 (s, 9H) ppm.

### Synthesis of N-(*tert*-Butoxycarbonyl)*-N,N*-bis(acryloxyethyl)amine

The synthesis was carried out according to a modified protocol from Xun et al.^40^*N*-(*tert*-butoxycarbonyl)diethanolamine (9.1 g, 1 equiv) was dissolved in anhydrous DCM (100 mL), and triethylamine (13.6 mL, 2.2 equiv) was added. The solution was cooled to 0 °C and acryloyl chloride (7.9 mL, 2.2 equiv) was added dropwise. The reaction was stirred for 1 h at 0 °C and afterward at room temperature overnight. Subsequently, the organic phase was washed with saturated NaHCO_3_ (5 × 100 mL) and dried over MgSO_4_. The solvent was removed by reduced pressure, and the crude product was purified via column chromatography (silica, EtOAc/hexane 1:3, *R*_f_ = 0.6) to obtain a colorless oil (10.9 g, 82%).

^1^H NMR (300 MHz, CDCl_3_, δ): 6.43 (d, *J* = 17.8 Hz, 2H), 6.13 (dd, *J* = 17.3, 10.4 Hz, 2H), 5.86 (dd, *J* = 10.6, 4.4 Hz, 2H), 4.29 (p, *J* = 5.5 Hz, 4H), 3.55 (dt, *J* = 12.4, 5.6 Hz, 4H), 1.46 (s, 9H) ppm.

### Synthesis of *N,N*-Bis(acryloxyethyl)amine (BAA)

The synthesis was carried out according to a protocol from Xun et al.^39^*N*-(*tert*-Butoxycarbonyl)*-N,N*-bis(acryloxyethyl)amine (2 g, 1 equiv) was dissolved in anhydrous DCM (20 mL) and cooled to 0 °C. TFA (3.3 mL, 6.75 equiv) was added dropwise, and the reaction was stirred for 3 h at room temperature. Afterward, the solvent was removed with reduced pressure. The crude product was dissolved in anhydrous DCM (50 mL), and diluted NH_3_ (2 mL, 25%) was added. The organic phase was separated and dried over MgSO_4_. Afterward, the solvent was removed by reduced pressure to obtain the product as a colorless oil (1.2 g, 78%). To prolong the shelf life, the compound was stored at −20 °C under an argon atmosphere.

^1^H NMR (300 MHz, CDCl_3_, δ): 6.43 (dd, *J* = 17.3, 1.5 Hz, 2H), 6.15 (dd, *J* = 17.3, 10.4 Hz, 2H), 5.86 (dd, *J* = 10.4, 1.5 Hz, 2H), 4.36–4.25 (m, 4H), 3.03–2.93 (m, 4H), 1.93 (s, 1H) ppm.

### Synthesis of *N*-Allyl-3-bromo-3-methylbutanamide (ABMP)

ABMP was synthesized as described before.^36^ Allylamine (3.59 mL, 1.1 equiv) was dissolved in dry dichloromethane (100 mL) and triethylamine (20 mL). Then, α-bromoisobutyryl bromide (5.38 mL, 1 equiv) was added dropwise under stirring at 0 °C. Afterward, the reaction was stirred for 30 min at 0 °C and subsequently for 5 h at room temperature. The mixture was filtered, and the filtrate was washed with NaHCO_3(aq.)_, water, and brine (3 × 100 mL each). After drying over MgSO_4_, the solvent was evaporated under reduced pressure. The oily product was dried *in vacuo* (7.8 g, 87%).

^1^H NMR (300 MHz, CDCl_3_, δ): 6.80 (s, 1H), 5.95–5.76 (m, 1H), 5.22–5.11 (m, 1H), 3.89 (tt, *J* = 5.7, 1.7 Hz, 2H), 1.97 (s, 6H) ppm.

### Synthesis of [PEG_26_-SH]_3_[PEG_26_-ABMP]

[PEG_26_-SH]_3_[PEG_26_-ABMP] was synthesized as described before.^36^ [PEG_26_-SH]_4_ (2.0 g, 1 equiv), ABMP (83.13 mg, 1 equiv), and DMPA (10.34 mg, 0.1 equiv) were dissolved in chloroform (8 mL) and stirred for 2 min under UV irradiation. Afterward, the polymer was precipitated in cold diethyl ether (200 mL) and washed with diethyl ether (3 × 60 mL). The polymer was dried under vacuum to obtain [PEG_26_-SH]_3_[PEG-ABMP] as a white solid (1.9 g, 92%).

^1^H NMR (300 MHz, D_2_O, δ): 3.72 (m, 432H), 3.35 (t, *J* = 6.7 Hz, 2H), 2.75 (m, 8H), 2.64 (t, *J* = 7.4 Hz, 2H), 1.95 (s, 6H), 1.90–1.78 (m, 2H) ppm.

SEC (DMAc + 0.21 wt % LiCl, PEG calibration): *M*_n_ = 4500 g mol^–1^, *M*_w_ = 5000 g mol^–1^, *Đ* = 1.1.

### Synthesis of [PEG_26_-SAc]_3_[PEG_26_-ABMP]

[PEG_26_-SAc]_3_[PEG_26_-ABMP] was synthesized as described before.^36^ [PEG_26_-SH]_3_[PEG_26_-ABMP] (1.8 g, 1 equiv) was dissolved in dry dichloromethane (35 mL), and triethylamine (0.58 mL, 12 equiv) was added. Acetyl chloride (0.30 mL, 12 equiv) was added dropwise at 0 °C, and the reaction was stirred for 30 min at 0 °C. Afterward, it was stirred for an additional 3 h at room temperature, and subsequently, the solvent was partially removed by reduced pressure. The polymer was precipitated in cold diethyl ether (200 mL) and washed with diethyl ether (3 × 30 mL). The crude product was dried under vacuum and further purified by dialysis against H_2_O (MWCO 1000 g mol^–1^). After freeze-drying, a white powder was obtained (1.1 g, 60%).

^1^H NMR (300 MHz, D_2_O, δ): 3.69 (s, 432H), 3.20 (t, *J* = 7.3 Hz, 2H) 3.12 (t, *J* = 6.2 Hz, 6H), 2.77 (t, *J* = 6.3 Hz, 2H), 2.62 (t, *J* = 7.3 Hz, 1H), 2.38 (s, 9H), 1.93 (s, 6H), 1.83 (t, *J* = 7.0 Hz, 2H) ppm.

SEC (DMAc + 0.21 wt % LiCl, PEG calibration): *M*_n_ = 5 100 g mol^–1^, *M*_w_ = 6 200 g mol^–1^, *Đ* = 1.2.

### Synthesis of [PEG_26_-SAc]_3_[PEG_26_-*b*-PDMAPAam_62_]

[PEG_26_-SAc]_3_[PEG_26_-ABMP] (1.0 g, 1 equiv), Me_6_TREN (25,26 μL, 0.5 equiv), and DMAPAam (2.95 g, 100 equiv) were dissolved in a mixture of H_2_O and *iso*-propanol (1:5 v/v, 36.8 mL) and a stock solution of CuCl_2_ (2.54 mg mL^–1^, 1 mL, 0.1 equiv) in the solvent mixture was added. Subsequently, the reaction mixture was degassed by four freeze–pump–thaw cycles. Cu (3.0 mg, 0.25 equiv) was added under an argon stream and the reaction was stirred for 24 h at room temperature and subsequently quenched by freezing and purging with air. Then, the solution was dialyzed against H_2_O (MWCO 1000 g mol^–1^) and freeze-dried. After freeze-drying, the polymer was obtained as a slightly yellow powder (2.0 g, 83%).

^1^H NMR (300 MHz, D_2_O, δ): δ 3.61 (s, 432H), 3.07 (s, 98H), 2.28 (s, 98H), 2.13 (s, 282H), 1.60 (s, 98H) ppm.

SEC (DMAc + 0.21 wt % LiCl, PEG calibration): *M*_n_ = 7000 g mol^–1^, *M*_w_ = 9 800 g mol^–1^, *Đ* = 1.4.

### Synthesis of [PEG_26_-SH]_3_[PEG_26_-*b*-PDMAPAam_62_]

[PEG_26_-SAc]_3_[PEG_26_-*b*-PDMAPAam_62_] (1.2 g) were dissolved in NaOH_aq._ (20 mL, 0.1 M) and stirred at room temperature for 1 h. Then, EDTA (584 mg, 0.1 M) was added to the solution, and the mixture was stirred for 1 h. The solution was dialyzed against H_2_O (MWCO 1000 g mol^–1^). After freeze-drying, the asymmetric block copolymer was obtained as a yellow solid (0.9 g, 75%).

^1^H NMR (300 MHz, D_2_O, δ): 3.70 (s, 432H), 3.21 (s, 98H), 2.90 (s, 98H), 2.68 (s, 282H), 1.87 (98 H) ppm.

### Synthesis of the PDMAPAam Hydrogel

In a general procedure, [PEG_26_-SH]_4_ (84.57 mg) and [PEG_26_-SH]_3_[PEG_26_-*b*-PDMAPAam_62_] (15.43 mg, 10 wt %) were dissolved in a 1 mM TCEP·HCl solution (in PBS, 1 mL) to obtain a total polymer concentration of 100 mg mL^–1^. The polymer solution was kept in the ultrasonification bath for 20 min. Afterward, TEG-DV (8.24 mg) and LAP (2.00 mg, 2 wt %) were dissolved in the polymer solution. Then, the mixture was irradiated under UV light for 1 min to form the respective hydrogel.

### Synthesis of BAA Hydrogels

In a general procedure, [PEG_26_-SH]_4_ (100 mg, 1 equiv) and BAA (8.53 mg, 2 equiv) were separately dissolved in PBS (2 × 0.5 mL). The solutions were mixed and cured for 20 min at room temperature.

### Synthesis of Mixed PDMAPAam/BAA Hydrogels

In a general procedure, [PEG_26_-SH]_4_ (84.57 mg) and [PEG_26_-SH]_3_[PEG_26_-*b*-PDMAPAam_62_] (15.43 mg, 10 wt %) were dissolved in 1 mM TCEP·HCl solution (in PBS, 0.5 mL). The polymer solution was kept in the ultrasonification bath for 20 min. Afterward, the solution was mixed with TEG (3.26 mg) and LAP (1.00 mg, 1 wt %). The resulting solution was mixed with a solution of BAA (8.53 mg; BAA/TEG 6:4) in 1 mM TCEP·HCl (in PBS, 0.5 mL) and cured for 1 min under UV irradiation.

### Swelling Experiments

For the determination of the swelling ratio, the hydrogel pre-solution was synthesized as described before. The determination was carried out with five replicates and a volume of 150 μL for each sample. The gel was formed in a disklike mold with a diameter of 8 mm. After gelation, the hydrogels were immersed in 3 mL of PBS each. For the determination of the weight, the surface water was carefully removed by wipes. Afterward, the hydrogels were freeze-dried, and the weight of the dry hydrogels was determined. The swelling degree was calculated with the following equation
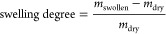


In the case of the normalized swelling, no dry weight was determined, but the initial weight of the sample prior to swelling was set to 1.

### Rheology

For the determination of the storage and loss modulus, the hydrogel pre-solution was synthesized as described before. For a planar sample, 1.2 mL of solution was filled between two glass slides with a 1 mm gap, and after curing for 20 min at room temperature, a disc of 20 mm diameter was cut out and swelled for 48 h. In the case of the degradation kinetics, the storage modulus was determined once per day until the hydrogels lost their mechanical integrity. The determination was carried out with triplicates. The measurements were carried out with a parallel plate measuring system with a diameter of 20 mm at 23.5 °C. Time-dependent experiments were carried out with a constant shear amplitude of 1%, a frequency of 6.28 rad·s^–1^, and a normal force of 1 N.

### Adsorption of Growth Factors

For the adsorption of TGF-β, the hydrogels were synthesized as described before. Before gelation, 240 μL of the solution was filled in a 24-well plate. The specimens were swollen in PBS (2 mL) for 48 h, with regular exchange of the solution every 12 h. Subsequently, the hydrogels were immersed in a Vitronectin solution (10 μg·mL^–1^ in PBS, 0.5 mL) with or without TGF-β (50 μg·mL^–1^) for 3 days.

### Cell Culture

3T3-JS cells were cultivated in DMEM containing 1 g/L glucose, 10% FBS, 1% glutamax, and 1% penicillin/streptomycin in T75 cell culture flasks up to 80% confluency. LX-2 cells were cultivated in DMEM containing 1 g/L glucose, 1% FBS, 1% glutamax, 1% pyruvate, and 1% penicillin/streptomycin in T25 flasks up to 80% confluency. Confluent flasks were split by use of a 0.25% trypsin/EDTA solution and seeded in a density of 100,000 cells per well and the hydrogel on a 24-well plate. For fluorescence microscopy, cells were fixated with methanol for 15 min at −20 °C and stained for 60 min at room temperature.

## Supporting Information Available

^1^H NMR spectra (300 MHz, δ in ppm) of *N*-(*tert*-butoxycarbonyl)diethanolamine, *N*-(*tert*-butoxycarbonyl)*-N,N*-bis(acryloxyethyl)amine, and BAA, ^1^H NMR spectra (300 MHz, δ in ppm) of [PEG_26_-SH]_3_[PEG_26_-ABMP] and [PEG_26_-SAc]_3_[PEG_26_-ABMP], and ^1^H NMR spectra (300 MHz, δ in ppm) of [PEG_26_-SAc]_3_[PEG_26_-*b*-PDMAPAam_62_] and [PEG_26_-SH]_3_[PEG_26_-*b*-PDMAPAam_62_] (PDF)
