# Supplementary material for: Tailoring the Degradation Time of Polycationic PEG-Based Hydrogels toward Dynamic Cell Culture Matrices
Source: ACS Appl Bio Mater. 2024 Mar 12;7(4):2402–12. doi: 10.1021/acsabm.4c00057 (PMC11022240; doi:10.1021/acsabm.4c00057)
Supplement: Supplementary file 1 — mt4c00057_si_001.pdf [file mt4c00057_si_001.pdf]

Supporting Information

Tailoring the degradation time of polycationic PEG-based hydrogels towards dynamic cell culture matrices

Kathrin Kowalczuk,[1,2,3] Valentin D. Wegner,[4] Alexander S. Mosig,[3,4,5] Felix H. Schacher[1,2,3*]

[1] Institute of Organic Chemistry and Macromolecular Chemistry (IOMC), Friedrich-Schiller-University Jena, Lessingstraße 8, D-07743 Jena, Germany

[2] Jena Center for Soft Matter (JCSM), Friedrich-Schiller-University Jena, Philosophenweg 7, D-07743 Jena, Germany

[3] Cluster of Excellence Balance of the Microverse, Friedrich Schiller University Jena, Grüne Aue, D-07754 Jena, Germany

[4] Institute of Biochemistry II, Jena University Hospital, Am Nonnenplan 2-4, 07743 Jena, Germany

[5] Center for Sepsis Control and Care, Jena University Hospital, Am Klinikum 1, 07747 Jena, Germany

Email: felix.schacher@uni-jena.de; alexander.mosig@med.uni-jena.de

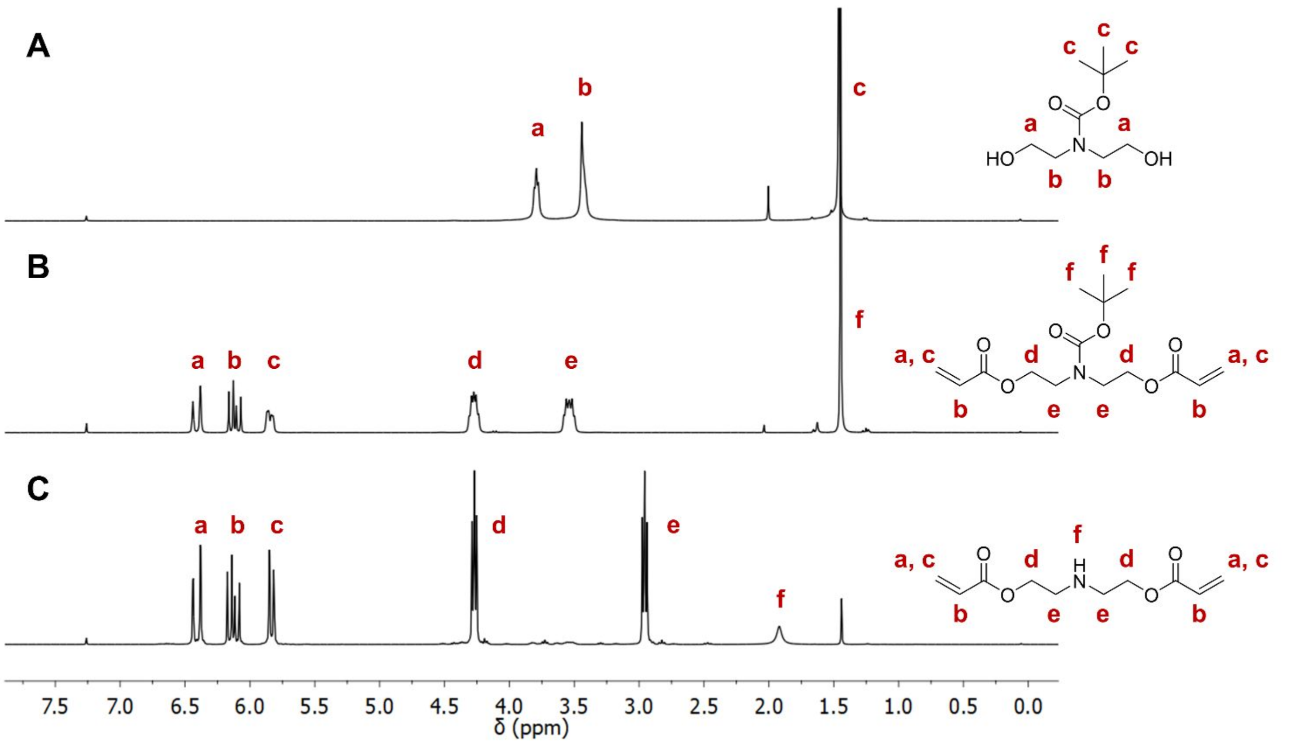

Figure S1: ^{1}H-NMR spectra (300 MHz, δ in ppm) of *N*-(*tert*-butoxycarbonyl)diethanolamine (A), N-(*tert*-butoxycarbonyl)-*N,N*-bis(acryloxyethyl)amine (B) and BAA (C) in $CDCl_3$.

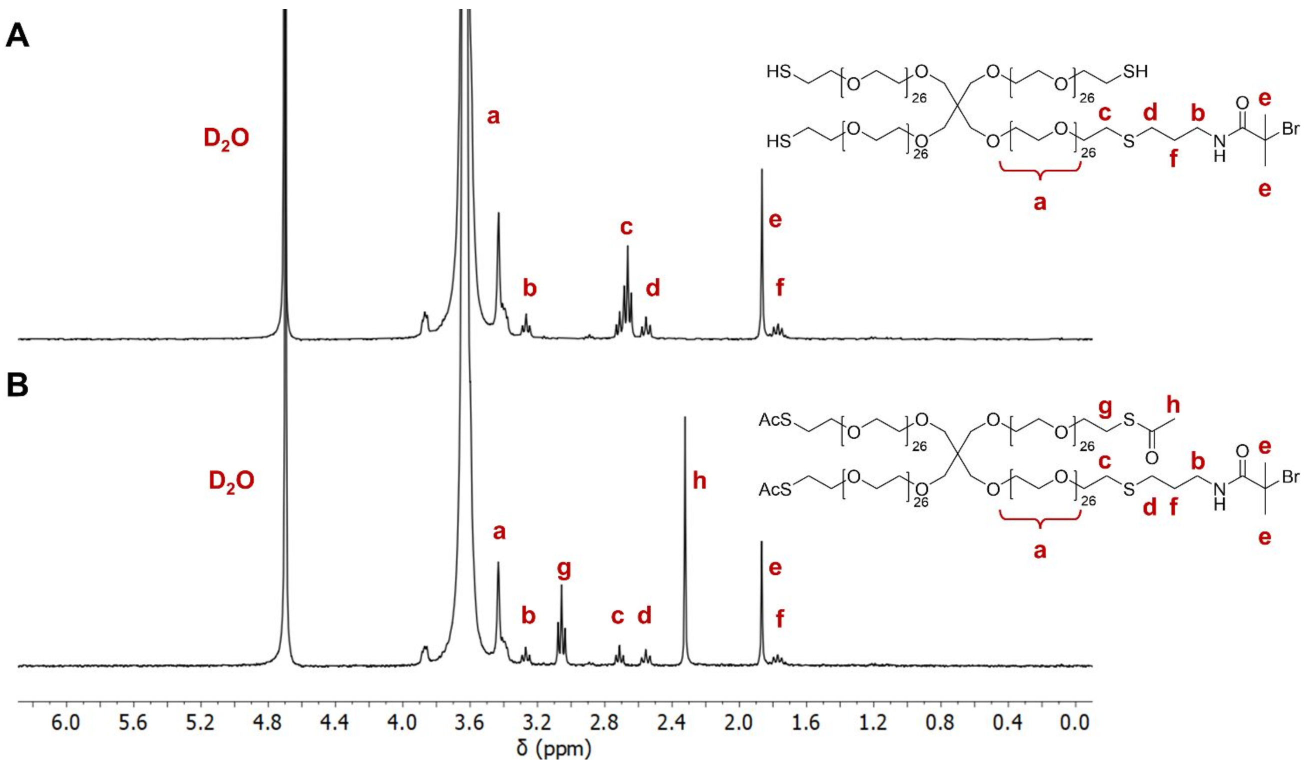

Figure S2: ^{1}H-NMR spectra (300 MHz, δ in ppm) of [PEG_{26}-SH]$_3$[PEG_{26}-ABMP] (A) and [PEG_{26}-SAc]$_3$[PEG_{26}-ABMP] (B) in D_2O.

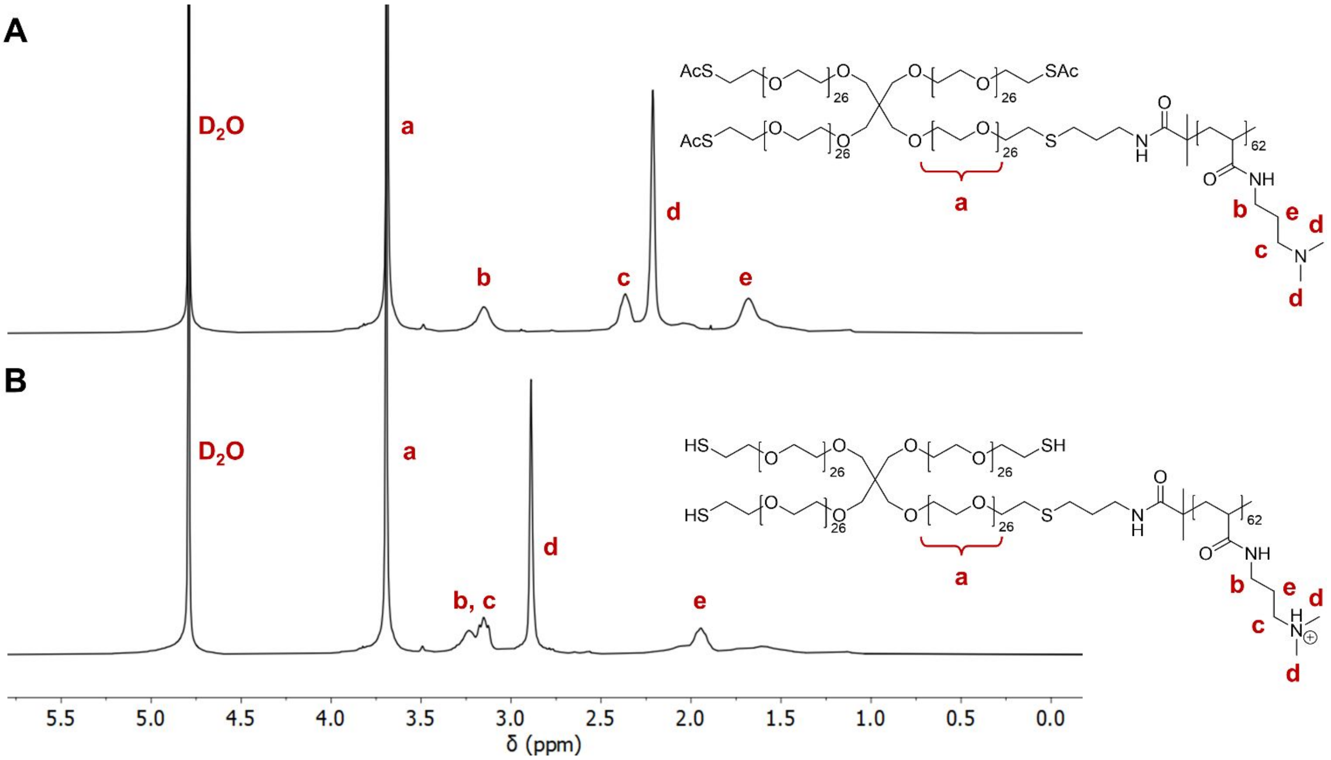

Figure S3: ^{1}H-NMR spectra (300 MHz, δ in ppm) of $[PEG_{26}\text{-}SAc]_3[PEG_{26}\text{-}b\text{-}PDMAPAam_{62}]$ (A) and $[PEG_{26}\text{-}SH]_3[PEG_{26}\text{-}b\text{-}PDMAPAam_{62}]$ (B) in D_2O.
